# Astrocytic connexin 43‐hemichannels aggravate seizures by modulating blood‐brain barrier permeability in temporal lobe epilepsy mice

**DOI:** 10.1002/pdi3.2500

**Published:** 2024-08-02

**Authors:** Honghong Song, Yi Li, Jiayu Liu, Xianshu Bai, Li Jiang, Tingsong Li

**Affiliations:** ^1^ Department of Neurology Children’s Hospital of Chongqing Medical University, National Clinical Research Center for Child Health and Disorders Ministry of Education Key Laboratory of Child Development and Disorders Chongqing Key Laboratory of Child Neurodevelopment and Cognitive Disorders Chongqing China; ^2^ Department of Rehabilitation Children’s Hospital of Chongqing Medical University, National Clinical Research Center for Child Health and Disorders Ministry of Education Key Laboratory of Child Development and Disorders Chongqing Key Laboratory of Child Neurodevelopment and Cognitive Disorders Chongqing China; ^3^ Molecular Physiology Center for Integrative Physiology and Molecular Medicine (CIPMM) University of Saarland Homburg Germany

**Keywords:** astrocytes, blood‐brain barrier, Cx43, epilepsy, hippocampus

## Abstract

The involvement of astrocytic connexin 43 (Cx43) in epileptogenesis has been extensively studied through various approaches, yet the underlying mechanism remains enigmatic. In this study, we explored whether astrocytic Cx43 forms hemichannels (HCs) that contribute to seizure progression in temporal lobe epilepsy (TLE) in mice. We focused on how these HCs influence the permeability of the blood‐brain barrier (BBB), a crucial factor in the pathophysiology of epilepsy. Immunofluorescence staining and western blot analysis were employed to assess Cx43 expression in kainic acid‐induced TLE mice, while BBB permeability was evaluated in TLE mice and those treated with TAT‐Gap19 (an astrocytic Cx43 HC inhibitor) using Evans Blue permeation, serum S100β protein quantification, ZO‐1 expression, and albumin extravasation into brain parenchyma via western blotting. Furthermore, seizure burden was monitored continuously using telemetric electroencephalography (EEG) and video monitoring in epileptic and TAT‐Gap19‐treated mice. Results demonstrated a significant increase in Cx43 content in hippocampal tissue in the TLE group, with a pronounced expression around blood vessels. TAT‐GAP19 treatment alleviated EEG seizures and BBB permeability in TLE mice. These findings suggest that astrocytic Cx43 HCs in the hippocampus play a crucial role in epileptogenesis and seizure progression by regulating BBB permeability. Targeting Cx43‐formed HCs distributed around the neurovascular unit may offer a novel therapeutic approach for epilepsy.

## Graphical abstract

The alterations of connexin 43 (Cx43) hemichannels contribute to the progress of seizures by regulating BBB permeability in temporal lobe epilepsy (TLE). Up‐regulated Cx43 accumulated on the astroglial endfeet in the hippocampi of TLE that functioned as hemichannels and thereby disrupted blood‐brain barrier (BBB) integrity. Inhibiting the Cx43‐hemichannels by TAT‐Gap19 reduced the BBB permeability and consequently relieved seizure severity, which may be associated with reduced release of adenosine triphosphate, glutamate and Ca^2+^ through Cx43 hemmichannels.

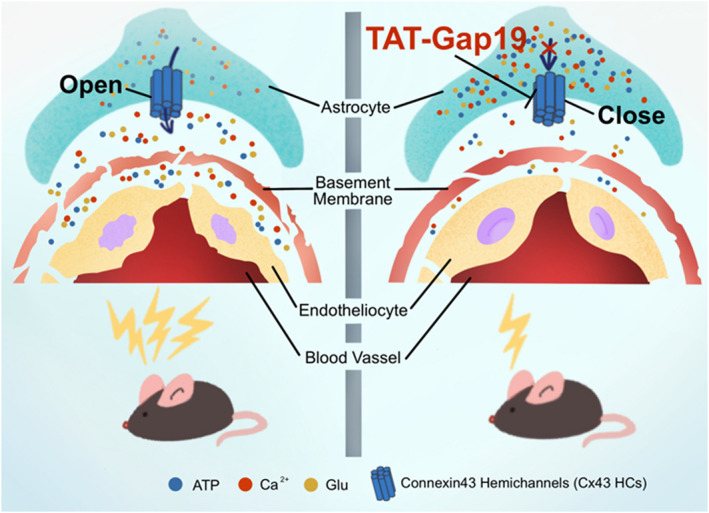

## 1 INTRODUCTION

Temporal lobe epilepsy (TLE), characterized by recurrent and spontaneous seizures, is the most prevalent form of epilepsy, frequently leading to neurobiological, cognitive, and psychosocial impairments.^1^ Despite the availability of multiple antiseizure medications targeting neuronal voltage‐gated ion channels and facilitating gamma‐aminobutyric acid (GABA)‐ergic inhibition, approximately 30% of patients remain refractory to pharmacological treatments.^2^ Accumulating evidence suggests that astrocytes play a crucial role in epilepsy onset and progression.^3^ As such, elucidating the dysfunction of these cells in seizure initiation and advancement could facilitate the development of novel treatment strategies for epilepsy. Connexin 43 (Cx43), the predominant connexin isoform in astrocytes, is integral in forming gap junctions and HCs on the cellular membrane. These gap junctions, formed by the docking of HCs from adjacent cells, enable the direct exchange of ions and small molecules (<1000 Da) between cells. HCs also regulate intracellular and extracellular communication by allowing the release of small molecules and ions into the extracellular space.^4^ Elevated levels of Cx43 have been reported in both human temporal epilepsy and TLE animal models.^5^ Our previous study revealed a pronounced subcellular redistribution of Cx43 towards perivascular endfeet, accompanied by blood‐brain barrier (BBB) damage and albumin extravasation in TLE mouse models.^5^ However, the precise mechanisms by which elevated and redistributed Cx43 influences the initiation and progression of seizures remain unclear. Studies have indicated that Cx43 can induce BBB hyperpermeability through its associated gap junctions and HCs in conditions such as familial cerebral cavernous malformations type III.^6^ Given the essential function of astrocytic endfeet in preserving BBB integrity, we propose that Cx43‐associated HCs located around the neurovascular unit are critically involved in the epileptogenesis and escalation of seizures by regulating BBB permeability. Targeting these HCs may provide a novel therapeutic avenue for epilepsy. In the current study, we investigated the role of astrocytic Cx43‐formed HCs in seizure progression in a mouse model of TLE. Combined immunohistochemical, molecular, and electrophysiological approaches were applied to assess Cx43 expression, BBB permeability, seizure burden, and associated molecular mechanisms. Our findings provide novel insights into the pathogenesis of epilepsy and pave the way for the development of more effective epilepsy treatments.

## 2 MATERIALS AND METHODS

### 2.1 Mouse model of TLE

C57BL/6 male mice were purchased from the Animal Resources Center of Chongqing Medical University, China. Eight‐week‐old age mice were used in all experiments. The mice were reared in a specific pathogen‐free environment under a light/dark period of 12 h at a temperature of 25 ± 2°C and relative humidity of 50% ± 5%. A mouse model of TLE induced by kainic acid (KA) was established, as described in previous research.^7^ Initially, the mice were intraperitoneally injected with KA (30 mg/kg, Cayman Chemical Company) to induce status epilepticus (SE). Upon reaching a seizure score >4 on the Racine scale, lasting 30 min, diazepam (0.5 mg/kg, Kingyork Group) was injected intraperitoneally to terminate SE. The control group received only 0.9% sodium chloride solution and diazepam at the corresponding time points. Mice that failed to develop SE or died subsequent to SE were excluded from the study. Surviving mice exhibiting spontaneous seizures 4 weeks after KA administration were classified as TLE mice. All animal experiments were approved by the Animal Ethics Committee of Chongqing Medical University and complied with its guidelines. Throughout the experiment, we used 70 C57BL/6 male mice to induce the TLE model with a mortality rate of approximately 15%.

### 2.2 Immunofluorescence staining

Mice were anesthetized using an intraperitoneal injection of pentobarbital sodium. Brain tissue was subjected to internal and external fixation using 4% paraformaldehyde and dehydration. Subsequently, brains were sliced into 30‐μm‐thick coronal frozen sections using a freezing rotary microtome. After high‐temperature repair and blocking, the sections were incubated with primary antibodies overnight at 4°C, followed by the application of suitable secondary antibodies and 4',6‐Diamidino‐2‐phenylindole back‐staining in the dark. Images were captured using a confocal microscope. The following primary antibodies were used: rabbit anti‐Cx43 polyclonal antibody (1:2500, Abcam, ab11370); rabbit anti‐CD31 polyclonal antibody (1:200, Zen‐Bioscience, R10021); rabbit anti‐Albumin monoclonal antibody (1:2000, Abcam, ab207327); mouse anti‐glial fibrillary acidic protein (GFAP) monoclonal antibody (1:5000, Abcam, ab279290).

### 2.3 Evans blue dye

To assess BBB integrity, Evans blue dye (EBD) was used to measure leakage into the brain, following the previously reported protocol.^8^ In brief, 2% EBD solution was injected into the tail veins of mice (3 mL/kg, Sigma). After 2 h, their brains were extracted, weighed, and incubated in 2 mL of N, N‐dimethylformamide for 24 h at 60°C. The amount of extracted dye was measured spectrophotometrically at 620 nm.

### 2.4 Enzyme‐linked immunosorbent assay

Serum samples were utilized to detect S100β levels. Samples were added to Enzyme‐linked immunosorbent assay (ELISA) plates as per the instructions provided by commercial ELISA kits (Jianglai Biology). Following incubation for 90 min at 37°C, the plates were washed and incubated with a horseradish peroxidase‐conjugated antibody for 60 min at 37°C. S100β concentrations were measured using a microplate spectrophotometer (Rayto RT‐6100) by detecting absorbance at 450 nm.

### 2.5 Western blotting

Total protein from the hippocampal tissue was obtained using a protein extraction kit (Keygen Biotech Corp., Ltd.). Cx43, primarily expressed in the cellular membrane, was extracted using a membrane protein extraction kit (Keygen Biotech Corp., Ltd.,). Following quantification of protein concentration using the bicinchoninic acid method, the protein was separated by 10% and 7.5% polyacrylamide gel electrophoresis and transferred onto polyvinylidene fluoride (PVDF) membranes (#1620177, Bio‐Rad). The membrane was blocked for 1 h at room temperature in phosphate‐buffered saline containing 5% bovine serum albumin and further incubated with specific primary detection antibodies overnight at 4°C. Subsequently, peroxidase‐conjugated secondary antibodies were applied to the same membranes for another 1 h at room temperature. Protein bands were visualized using clarity western electrochemiluminescence substrate (Bio‐Rad) and analyzed by densitometry using Image J software (NIH). The following primary antibodies were used: rabbit anti‐Cx43 monoclonal antibody (1:2500, Abcam, ab11370); rabbit anti‐ZO‐1 polyclonal antibody (1:1000, ThermoFisher, 61‐7300); rabbit anti‐Albumin monoclonal antibody (1:2000, Abcam, ab207327); mouse anti‐β‐actin monoclonal antibody, (1:1000, Zen‐Bioscience; 700068); rabbit anti‐E‐cadherin monoclonal antibody (1:500, ABclonal, A20798).

### 2.6 TAT‐Gap19 administration

Considering Cx HC has low expression and a small probability of opening in cells of normal tissues,^9^ we introduced TAT‐Gap19 only in TLE mice to evaluate the influence of Cx43 HC on seizure propagation. The mice were randomly assigned to either the TLE control group or the TLE group treated with TAT‐Gap19 (TLE + TAT‐Gap19). The TLE + TAT‐Gap19 group was intraperitoneally injected with TAT‐Gap19 at a dose of 50 mg/kg (Macklin) on day 25 following KA‐induced SE and sacrificed 3 days later.

### 2.7 Seizure burden evaluation

Electrode implantation was performed according to previously described protocols.^10^ Seven days post‐implantation, 21 days after inducing SE, each animal (TLE or TLE + TAT‐Gap19) was transferred to an observation box and their electrodes were connected to an EEG100 C amplifier for video‐EEG recording for 2 h, with the electroencephalography (EEG) signals filtered at 0.1–500 Hz and digitalized at 1 kHz for subsequent analysis. Visual EEG analysis was conducted using LabChart 8 Reader (Australia). Seizure behaviors in all groups (TLE, *n* = 5; TLE + TAT‐Gap19, *n* = 5) were simultaneously evaluated with the EEG recordings. Seizures were classified based on the Racine scale^11^ as follows: “mouth and facial movements” (stage I); “head nodding” (stage II); “forelimb clonus” (stage III); seizures with rearing, (stage IV); and seizures with rearing and falling (stage V). Electrical seizures were defined according to specific criteria established in a previous study,^12^ including the presence of regular spike clusters lasting for more than 10 s, with a spike frequency exceeding 3 Hz and an amplitude at least three times higher than baseline.

### 2.8 Statistical analysis

Statistical analysis was conducted using GraphPad Prism (v9.2.0). Quantitative data were expressed as mean ± standard deviation (SD). Unpaired Student's *t*‐test was used to compare differences between two groups, while one‐way analysis of variance and Tukey‐Kramer *post hoc* test were employed for comparisons among more than two groups. Statistical significance was considered at *p < 0.05, **p < 0.01, ***p < 0.001, and ****p < 0.0001.

## 3 RESULTS

### 3.1 Increased Cx43 protein was predominantly distributed around blood vessels

**FIGURE 1:**
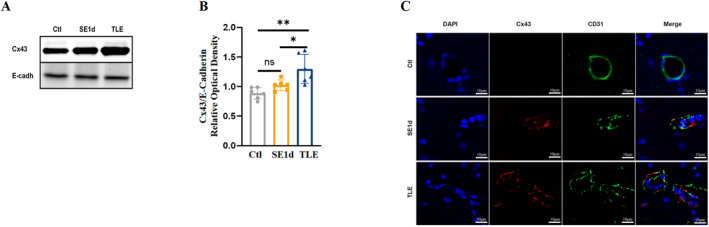
Increased Cx43 content and redistribution around blood vessels in the hippocampi in TLE mice. (A) Western blotting analysis showed membrane Cx43 protein levels at the SE1d group and TLE group, respectively. (B) Quantitative analyses of the Cx43 protein levels in the TLE group significantly increased compared to both the control group and SE1d group (*n* = 6). (C) Immunofluorescence staining of CD31 in green, Cx43 in red and DAPI in blue showed more Cx43 aggregates around blood vessels in the TLE group (scale bar = 10 μm, *n* = 4). All data are expressed as the mean ± SD, *p < 0.05, **p < 0.01. Cx43, connexin 43; TLE, temporal lobe epilepsy. DAPI, 4',6‐diamidino‐2‐phenylindole.

The results revealed a marked elevation in the expression of membrane‐bound Cx43 protein in TLE mice relative to the controls (Figure 1), with this up‐regulation becoming evident on day one post‐SE (SE1d) and intensifying in TLE conditions. Using E‐Cadherin as a loading control, western blot analysis showed a significant difference in Cx43 expression between the control and TLE mice (Figure 1A,B; *P*_*Ctl vs TLE*_ *<* 0.01, *P*_*SE1d vs TLE*_ *<* 0.05). E‐Cadherin was selected as a loading control due to its lack of association with epilepsy, ensuring a reliable baseline for assessing Cx43 expression. To examine the localization of the Cx43 protein, CD31 immunostaining was employed to delineate the vasculature. Double immunostaining of CD31/Cx43 revealed a marked increase in Cx43 plaques around blood vessels in both the SE1d and TLE groups compared to the control (Figure 1C), suggesting preferential localization of Cx43 at vascular interfaces in epileptic mice, potentially contributing to the pathogenesis of TLE. Together, these findings implicate Cx43 in the development and progression of epileptic pathology, particularly in the context of vascular mechanisms.

### 3.2 Hyperpermeability of BBB in TLE

**FIGURE 2:**
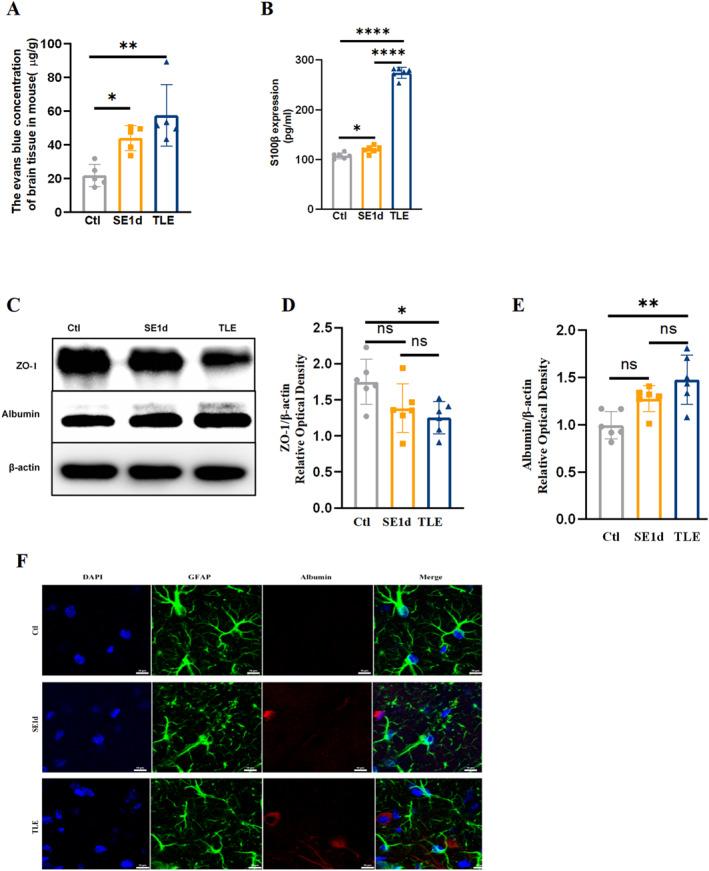
Increased BBB permeability in TLE mice. Evans blue content (*n* = 5, A), serum S100β content by ELISA (*n* = 6, B), and albumin protein levels in the brain by western blot (*n* = 6, E) were all increased significantly in the TLE group when compared to the control group (C), of which EBD and S100β became obviously elevated on SE1d. Quantitative analyses showed that ZO‐1 protein levels in the TLE group were significantly decreased compared to the control group (*n* = 6, C–D). (F) Double immunofluorescence staining showed the overlap of GFAP and albumin was obvious in the TLE group. (Scale bar = 10 μm, *n* = 4). All data are expressed as the mean ± SD, *p < 0.05, **p < 0.01, and ****p < 0.0001. BBB, blood‐brain barrier; EBD, Evans blue dye; GFAP, glial fibrillary acidic protein; TLE, temporal lobe epilepsy.

The evaluation of BBB permeability was conducted using EBD extravasation, ELISA for serum S100β levels, ZO‐1 and albumin western blotting, and albumin immunofluorescence staining (Figure 2). Results indicated a significant increase in EBD concentration in the SE1d and TLE groups compared to the control group (Figure 2A, *P*_*Ctl vs SE1d*_ *<* 0.05, *P*_*Ctl vs TLE*_ *<* 0.01). Serum S100β levels exhibited dynamic changes similar to the EBD results (Figure 2B, *P*_*Ctl vs SE1d*_ < 0.05, *P*_*Ctl vs TLE*_ *<* 0.0001, *P*_*SE1d vs TLE*_ *<* 0.0001). Western blot analysis of ZO‐1, a crucial protein for tight junction integrity and BBB permeability,^13^ revealed a marked reduction in expression in the TLE group (Figure 2C,D, *P*_*Ctl vs TLE*_ *<* 0.05). Assessment of albumin expression in the brain parenchyma by GFAP/albumin immunostaining and western blot analysis demonstrated a significant increase (Figure 2C,D,F,I, *P*_*Ctl vs TLE*_ *<* 0.01), with substantial co‐localization with GFAP‐labeled astrocytes (Figure 2F). Collectively, these findings suggest BBB impairment, resulting in albumin extravasation into the brain parenchyma.

### 3.3 BBB permeability in TLE was modulated by astrocytic Cx43 HC

**FIGURE 3:**
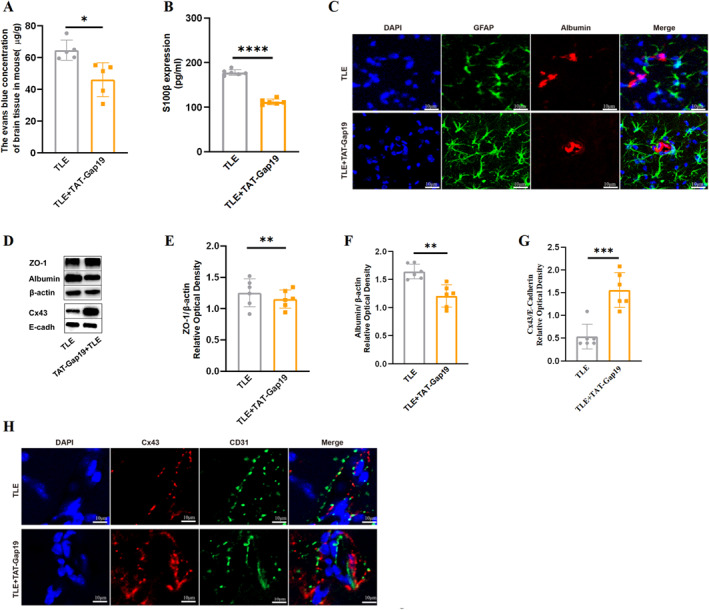
Improvement of BBB permeability after TAT‐Gap19 intervention in TLE mice. The changes in EBD content (*n* = 5, A), serum S100β by ELISA (*n* = 6, B), ZO‐1 (*n* = 6, D–E), and albumin (*n* = 6, D, F) content in hippocampi were all significant when treated with TAT‐Gap19. (C) Double immunofluorescence staining of GFAP and albumin showed less albumin leakage on GFAP + astrocytes in TLE + TAT‐Gap19. (Scale bar = 10 μm, *n* = 4). (G) Quantitative analyses of the membrane Cx43 protein levels in the TLE + TAT‐Gap19 group significantly increased compared to the TLE group (*n* = 6). (H) Double immunofluorescence staining of CD31/Cx43 showed still more Cx43 aggregates around blood vessels after TAT‐Gap19 treatment. (Scale bar = 10 μm, *n* = 4). All data are expressed as the mean ± SD, *p < 0.05, **p < 0.01, ***p < 0.001, ****p < 0.0001. BBB, blood‐brain barrier; EBD, Evans blue dye; GFAP, glial fibrillary acidic protein; TLE, temporal lobe epilepsy.

To investigate the role of Cx43 HCs in BBB permeability during TLE, TAT‐Gap19, a specific inhibitor of Cx43 HCs that does not interfere with gap junctions,^14^ was administered to TLE mice, with BBB permeability compared with non‐treated mice. Results showed that administration of TAT‐Gap19 significantly attenuated BBB disruption in TLE mice, as evident from EBD extravasation (Figure 3A, *P*_*TLE vs TLE + TAT‐Gap19*_ *<* 0.05), serum S100β concentration (Figure 3B, *P*_*TLE vs TLE + TAT‐Gap19*_ *<* 0.0001), Z0‐1 expression and albumin western blotting and immunostaining (Figure 3D,E, *P*_*TLE vs TLE + TAT‐Gap19*_ *<* 0.01; Figure 3D,F, *P*_*TLE vs TLE + TAT‐Gap19*_ *<* 0.01; Figure 3C). To exclude the possibility that the observed effects were due to altered Cx43 expression, Cx43 protein levels were compared between pre‐and post‐TAT‐Gap19 treatment in TLE mice. Immunostaining and western blotting revealed a significant increase in Cx43 expression (Figure 3D,G, *P*_*TLE vs*. *TLE + TAT‐Gap19*_ *<* 0.001) with maintained distribution around blood vessels (Figure 3H). These findings suggest that TAT‐Gap19 effectively mitigates BBB impairment in TLE mice without downregulation of Cx43 expression, thus implicating Cx43 HCs in the regulation of BBB permeability during epilepsy.

### 3.4 Improvement in seizures after astrocytic Cx43 HC inhibition

**FIGURE 4:**
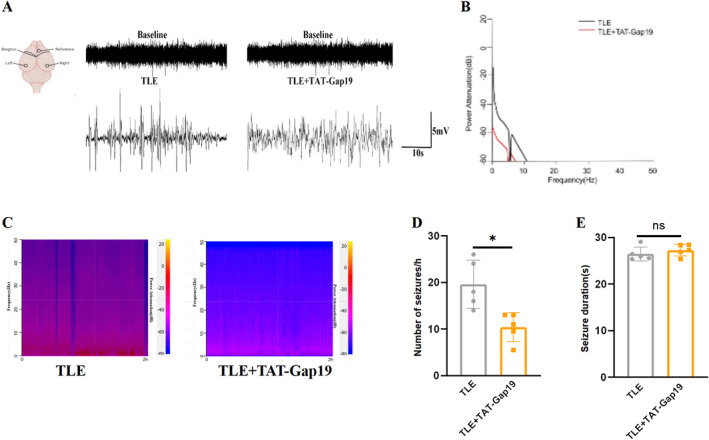
Seizure changes after TAT‐Gap19 intervention in TLE mice. (A) Representative EEG recordings in the TLE group and TLE + TAT‐Gap19 group in the ictal stage of spontaneous seizures. (B) Power spectral analysis of the TLE group and TLE + TAT‐Gap19 group. (C) Representative power spectrum density in the TLE group and TLE + TAT‐Gap19 group during the 2‐h EEG recordings. (D) TAT‐GAP19 reduced the frequency of spontaneous seizures in TLE mice (*n* = 5). (E) Effects of TAT‐Gap19 treatment on spontaneous seizure duration (seconds) (*n* = 5). All data are expressed as the mean ± SD, *p < 0.05. EEG, electroencephalography; TLE, temporal lobe epilepsy.

Continuous 2‐h EEG recordings were conducted to assess changes in seizure burden in TLE mice following treatment with TAT‐Gap19. As shown in Figure 4, TAT‐Gap19 treatment significantly reduced electrographic seizure activities, evident from both raw EEG traces and energy spectrogram analysis (Figure 4A,C). Power spectral analysis revealed that TAT‐Gap19 had a more pronounced effect on the power of low‐frequency bands compared to high‐frequency bands (Figure 4B). On day 28 after epilepsy induction, the frequency of spontaneous seizures was significantly lower in the TLE + TAT‐Gap19 group compared to the TLE group (Figure 4D, *P*_*TLE vs*. *TLE + TAT‐Gap19*_ *<* 0.05). However, no significant difference was observed in the mean duration of spontaneous seizures between the TLE and TLE + TAT‐Gap19 groups (Figure 4E, *P*_*TLE vs*. *TLE + TAT‐Gap19*_ > 0.05). These findings suggest that dysfunctional Cx43 HCs contribute to seizure progression in TLE, affecting primarily the frequency rather than the duration of seizures.

## 4 DISCUSSION

Our study indicated that increased Cx43 was involved in the disruption of BBB permeability at the early genesis of epilepsy, and that inhibition of astrocytic Cx43‐formed HCs improved BBB permeability and attenuated seizure burden. Considering that increased BBB permeability leads to albumin extravasation and leukocyte diapedesis from the peripheral blood into the brain parenchyma—a process that can trigger or exacerbate epileptogenesis^5^, ^15^—targeting astrocytic Cx43 HCs represents a promising approach for preventing the initiation and progression of epilepsy.

Astrocytic functions, including responses and communication with neighboring cells and the extracellular matrix, are mediated by Cx43‐formed gap junctions and HCs. Cx43/Cx43 and Cx43/Cx30‐formed gap junctions facilitate the intercellular flux of ions and small molecules, such as inositol trisphosphate, adenosine triphosphate (ATP), glutamate, and energy metabolites.^16^, ^17^ Deshpande et al. reported that the knockdown of both Cx30 and Cx43 in astrocytes results in a significant increase in seizure frequency, seizure burden, and EEG discharge.^18^ Bedner et al. revealed that astrocyte uncoupling due to Cx43‐formed gap junction dysfunction is a very early event in epileptogenesis, leading to impaired potassium clearance.^19^ In comparison, astrocytic HCs, predominantly hexamers of Cx43 located in astrocytic processes, permit the release of gliotransmitters and ion fluxes, thereby regulating hippocampal synaptic transmission and plasticity.^16^, ^17^ Walrave et al. found that Cx43 HC inhibition protects against seizures by lowering extracellular D‐serine levels.^20^ Furthermore, inhibition of connexin HCs alleviates neuroinflammation and hyperexcitability in TLE.^21^ These observations suggest that Cx43 is involved in seizure genesis through diverse mechanisms.

The BBB is a dynamic and complex barrier, essential for the normal functioning of the central nervous system and the maintenance of a regulated extracellular environment around synapses and axons.^22^, ^23^, ^24^ BBB dysfunction represents an important hallmark of brain seizures.^25^ Consistent with our previous study,^5^ we observed a pronounced distribution of increased Cx43 around blood vessels in TLE mice, suggesting potential participation in seizure propagation through disruption of BBB integrity. S100β is a calcium‐binding protein mainly concentrated in astrocytes in the nervous system.^26^ Serum S100β can be detected at very low levels under physiological conditions and increased content has been regarded as a surrogate marker for disrupted BBB integrity.^27^ In alignment with earlier research,^28^, ^29^ significant BBB permeability impairment in TLE mice was found in this study, that indicated by the marked alteration of serum S100β levels, the expression of ZO‐1 and albumin extravasation in hippocampi. The marked reduction in ZO‐1 levels in TLE was indicative of structural alterations in the BBB during seizure progression, given the essential function of ZO‐1 in tight junction protein assembly and integration into actin filaments within the brain.^30^ Astrocytic Cx43 HC activity is increased in pilocarpine‐induced epilepsy models, as evidenced by enhanced ethidium bromide uptake,^31^ potentially linked to elevated intracellular calcium (Ca^2+^) levels^32^ and post‐translational dephosphorylation.^33^ Given that various neurotransmitters, including ATP and glutamate,^34^ as well as neuroinflammation molecules^35^ can be released from Cx43 HCs, the opening of astrocytic Cx43 HCs may be involved in BBB impairment in TLE in various manners.

TAT‐Gap19, a specific inhibitor of Cx43 HCs that does not affect gap junctions, operates by binding to the carboxyl terminal, thereby preventing C‐terminus‐cytoplasmic loop intervention.^36^ As expected, treatment with TAT‐Gap19 markedly improved BBB permeability, evidenced by reductions in EBD, serum S100β, and albumin content, along with increased ZO‐1 expression. The results may be ascribed to the reductive release of glutamate, ATP and TNF‐α to the extracellular space interfaced against pericytes, endothelial cells of blood vessels, where they can induce BBB permeability through activation of N‐methyl‐D‐aspartate receptors,^37^ purinergic receptors^38^ and trigger neurovascular inflammation.^21^ Interestingly, we found that Cx43 was up‐regulated, rather than down‐regulated, when treated with TAT‐Gap19, excluding the impact of decreased Cx43 on BBB integrity. The reason for the increase in Cx43 levels post‐TAT‐Gap19 treatment is not clear but is speculated to be a compensatory response to the inhibition of HC function.

Although Cx43 HCs are expressed on capillary endothelial cells in the BBB,^39^ the mechanism by which Cx43 expression in endothelial cells is altered in TLE remains uncertain.^25^ Previous research has shown that Cx43 on the vascular endothelium affects BBB integrity through the Cx43‐PARP1 axis, rather than the classical channel approach.^40^ Therefore, Cx43 HCs on endothelial cells are unlikely to be involved in BBB permeability. Instead, the activation of Cx43 HCs in astrocytes during TLE may promote proconvulsive effects by impairing BBB integrity, rather than capillary endothelial cells, suggesting a diverse involvement in the genesis and progression of seizures.

## 5 CONCLUSIONS

In conclusion, during the chronic phase of epilepsy, Cx43 protein expression is up‐regulated and redistributed towards the perivascular region. This abnormal distribution of Cx43 is implicated in seizure progression in TLE by regulating BBB permeability via astrocytic Cx43 HCs accumulated on endfeet. While TAT‐Gap19 intervention alleviates BBB permeability in TLE, our mechanistic understanding of the abnormally distributed Cx43 and detailed processes involved in BBB disruption in TLE remains to be fully elucidated. Given their role in BBB regulation, astrocytic Cx43 HCs represent a potential novel target for the development of antiepileptic interventions.

## AUTHOR CONTRIBUTIONS

**Honghong Song**: Investigation; writing original draft preparation. **Yi Li**: Investigation; visualization. **Jiayu Liu**: Visualization; writing original draft preparation. **Xianshu Bai**: Writing – review & editing. **Li Jiang**: Conceptualization. **Tingsong Li**: Conceptualization; writing – review & editing.

## CONFLICT OF INTEREST STATEMENT

The authors declare no competing interests.

## ETHICS STATEMENT

The animal study protocol was approved by the Ethics Committee of Children's Hospital of ChongQing Medical University. (Approval number:CHCMU‐IACUC202206290‐05. Date of approval: 29 June 2020).

## Data Availability

The data that support the findings of this study are available from the corresponding author. The data are not publicly available due to ethical and privacy constraints.

## References

[1] Fisher RS , Acevedo C , Arzimanoglou A , et al. ILAE official report: a practical clinical definition of epilepsy. Epilepsia. 2014;55(4):475‐482. 24730690 10.1111/epi.12550

[2] Kwan P , Brodie MJ . Early identification of refractory epilepsy. N Engl J Med. 2000;342(5):314‐319. 10660394 10.1056/NEJM200002033420503

[3] Vezzani A , Ravizza T , Bedner P , Aronica E , Steinhäuser C , Boison D . Astrocytes in the initiation and progression of epilepsy. Nat Rev Neurol. 2022;18(12):707‐722. 36280704 10.1038/s41582-022-00727-5 PMC10368155

[4] Solan JL , Lampe PD . Src regulation of Cx43 phosphorylation and gap junction turnover. Biomolecules. 2020;10(12):1596. 33255329 10.3390/biom10121596 PMC7759836

[5] Deshpande T , Li T‐S , Herde MK , et al. Subcellular reorganization and altered phosphorylation of the astrocytic gap junction protein connexin43 in human and experimental temporal lobe epilepsy. Glia. 2017;65(11):1809‐1820. 28795432 10.1002/glia.23196

[6] Johnson AM , Roach JP , Hu A‐N , et al. Connexin 43 gap junctions contribute to brain endothelial barrier hyperpermeability in familial cerebral cavernous malformations type III by modulating tight junction structure. FASEB J. 2018;32(5):2615‐2629. 29295866 10.1096/fj.201700699R PMC5901390

[7] Rusina E , Bernard C , Williamson A . The kainic acid models of temporal lobe epilepsy. eNeuro. 2021;8(2): ENEURO.0337‐ENEURO.0320.2021. 10.1523/ENEURO.0337-20.2021 PMC8174050 33658312

[8] Wang Z‐F , Higashikawa K , Yasui H , et al. FTY720 protects against ischemia‐reperfusion injury by preventing the redistribution of tight junction proteins and decreases inflammation in the subacute phase in an experimental stroke model. Transl Stroke Res. 2020;11(5):1103‐1116. 32103462 10.1007/s12975-020-00789-x PMC7496052

[9] Orellana JA , Froger N , Ezan P , et al. ATP and glutamate released *via* astroglial connexin 43 hemichannels mediate neuronal death through activation of pannexin 1 hemichannels. J Neurochem. 2011;118(5):826‐840. 21294731 10.1111/j.1471-4159.2011.07210.x PMC3108012

[10] Zhang QJ , Zheng TG , Cheng T , Jia JY , Liang JH . Establishment of sub‐scalp electroencephalogram electrode embedding method in rats and mice (In Chinese). Chin J Pharm Toxicol. 2021;35(5):366‐373.

[11] Racine RJ . Modification of seizure activity by electrical stimulation: II. Motor seizure. Electroencephalogr Clin Neurophysiol. 1972;32(3):281‐294. 4110397 10.1016/0013-4694(72)90177-0

[12] Chen K‐N , Guan Q‐W , Yin X‐X , Wang Z‐J , Zhou H‐H , Mao X‐Y . Ferrostatin‐1 obviates seizures and associated cognitive deficits in ferric chloride‐induced posttraumatic epilepsy *via* suppressing ferroptosis. Free Radic Biol Med. 2022;179:109‐118. 34952157 10.1016/j.freeradbiomed.2021.12.268

[13] Furuse M , Tsukita S . Claudins in occluding junctions of humans and flies. Trends Cell Biol. 2006;16(4):181‐188. 16537104 10.1016/j.tcb.2006.02.006

[14] Wang N , de Vuyst E , Ponsaerts R , et al. Selective inhibition of Cx43 hemichannels by Gap19 and its impact on myocardial ischemia/reperfusion injury. Basic Res Cardiol. 2013;108(1):309. 23184389 10.1007/s00395-012-0309-x PMC3666173

[15] Zabrodskaya Y , Paramonova N , Litovchenko A , et al. Neuroinflammatory dysfunction of the blood‐brain barrier and basement membrane dysplasia play a role in the development of drug‐resistant epilepsy. Int J Mol Sci. 2023;24(16):12689. 37628870 10.3390/ijms241612689 PMC10454729

[16] Charvériat M , Naus CC , Leybaert L , Sáez JC , Giaume C . Connexin‐dependent neuroglial networking as a new therapeutic target. Front Cell Neurosci. 2017;11:174. 28694772 10.3389/fncel.2017.00174 PMC5483454

[17] Orellana JA , Retamal MA , Moraga‐Amaro R , Stehberg J . Role of astroglial hemichannels and pannexons in memory and neurodegenerative diseases. Front Integr Neurosci. 2016;10:26. 27489539 10.3389/fnint.2016.00026 PMC4951483

[18] Deshpande T , Li T‐S , Henning L , et al. Constitutive deletion of astrocytic connexins aggravates kainate‐induced epilepsy. Glia. 2020;68(10):2136‐2147. 32240558 10.1002/glia.23832

[19] Bedner P , Dupper A , Hüttmann K , et al. Astrocyte uncoupling as a cause of human temporal lobe epilepsy. Brain. 2015;138(Pt 5):1208‐1222. 25765328 10.1093/brain/awv067 PMC5963418

[20] Walrave L , Pierre A , Albertini G , et al. Inhibition of astroglial connexin43 hemichannels with TAT‐Gap19 exerts anticonvulsant effects in rodents. Glia. 2018;66(8):1788‐1804. 29683209 10.1002/glia.23341

[21] Guo A‐N , Zhang H‐Q , Li H‐H , et al. Inhibition of connexin hemichannels alleviates neuroinflammation and hyperexcitability in temporal lobe epilepsy. Proc Natl Acad Sci USA. 2022;119(45):e2213162119. 36322757 10.1073/pnas.2213162119 PMC9659366

[22] Obermeier B , Daneman R , Ransohoff RM . Development, maintenance and disruption of the blood‐brain barrier. Nat Med. 2013;19(12):1584‐1596. 24309662 10.1038/nm.3407 PMC4080800

[23] Abbott NJ , Patabendige AAK , Dolman DEM , Yusof SR , Begley DJ . Structure and function of the blood‐brain barrier. Neurobiol Dis. 2010;37(1):13‐25. 19664713 10.1016/j.nbd.2009.07.030

[24] Ballabh P , Braun A , Nedergaard M . The blood‐brain barrier: an overview: structure, regulation, and clinical implications. Neurobiol Dis. 2004;16(1):1‐13. 15207256 10.1016/j.nbd.2003.12.016

[25] Löscher W , Friedman A . Structural, molecular, and functional alterations of the blood‐brain barrier during epileptogenesis and epilepsy: a cause, consequence, or both? Int J Mol Sci. 2020;21(2):591. 31963328 10.3390/ijms21020591 PMC7014122

[26] Michetti F , Clementi ME , Di Liddo R , et al. The S100B protein: a multifaceted pathogenic factor more than a biomarker. Int J Mol Sci. 2023;24(11):9605. 37298554 10.3390/ijms24119605 PMC10253509

[27] Kawata K , Liu CY , Merkel SF , Ramirez SH , Tierney RT , Langford D . Blood biomarkers for brain injury: what are we measuring? Neurosci Biobehav Rev. 2016;68:460‐473. 27181909 10.1016/j.neubiorev.2016.05.009 PMC5003664

[28] van Vliet EA , Aronica E , Gorter JA . Blood‐brain barrier dysfunction, seizures and epilepsy. Semin Cell Dev Biol. 2015;38:26‐34. 25444846 10.1016/j.semcdb.2014.10.003

[29] Vazana U , Veksler R , Pell GS , et al. Glutamate‐mediated blood‐brain barrier opening: implications for neuroprotection and drug delivery. J Neurosci. 2016;36(29):7727‐7739. 27445149 10.1523/JNEUROSCI.0587-16.2016 PMC4951577

[30] van Lanen RH , Melchers S , Hoogland G , et al. Microvascular changes associated with epilepsy: a narrative review. J Cerebr Blood Flow Metabol. 2021;41(10):2492‐2509. 10.1177/0271678X211010388 PMC8504411 33866850

[31] Cheung G , Bataveljic D , Visser J , et al. Physiological synaptic activity and recognition memory require astroglial glutamine. Nat Commun. 2022;13(1):753. 35136061 10.1038/s41467-022-28331-7 PMC8826940

[32] Carmignoto G , Haydon PG . Astrocyte calcium signaling and epilepsy. Glia. 2012;60(8):1227‐1233. 22389222 10.1002/glia.22318 PMC4532388

[33] Purnell BS , Alves M , Boison D . Astrocyte‐neuron circuits in epilepsy. Neurobiol Dis. 2023;179:106058. 36868484 10.1016/j.nbd.2023.106058 PMC10334651

[34] Chandrasekhar A , Bera AK . Hemichannels: permeants and their effect on development, physiology and death. Cell Biochem Funct. 2012;30(2):89‐100. 22392438 10.1002/cbf.2794

[35] Lei L , Wang Y‐T , Hu D , Gai C , Zhang Y . Astroglial connexin 43‐mediated gap junctions and hemichannels: potential antidepressant mechanisms and the link to neuroinflammation. Cell Mol Neurobiol. 2023;43(8):4023‐4040. 37875763 10.1007/s10571-023-01426-5 PMC11407732

[36] Delvaeye T , Vandenabeele P , Bultynck G , Leybaert L , Krysko DV . Therapeutic targeting of connexin channels: new views and challenges. Trends Mol Med. 2018;24(12):1036‐1053. 30424929 10.1016/j.molmed.2018.10.005

[37] Xhima K , Weber‐Adrian D , Silburt J . Glutamate induces blood‐brain barrier permeability through activation of N‐methyl‐D‐aspartate receptors. J Neurosci. 2016;36(49):12296‐12298. 27927949 10.1523/JNEUROSCI.2962-16.2016 PMC6601974

[38] Wang Y‐M , Zhu Y‐B , Wang J‐M , et al. Purinergic signaling: a gatekeeper of blood‐brain barrier permeation. Front Pharmacol. 2023;14:1112758. 36825149 10.3389/fphar.2023.1112758 PMC9941648

[39] Tachikawa M , Murakami K , Akaogi R , Akanuma SI , Terasaki T , Hosoya KI . Polarized hemichannel opening of pannexin 1/connexin 43 contributes to dysregulation of transport function in blood‐brain barrier endothelial cells. Neurochem Int. 2020;132:104600. 31712070 10.1016/j.neuint.2019.104600

[40] Zhan R , Meng X , Tian D‐P , et al. NAD^+^ rescues aging‐induced blood‐brain barrier damage *via* the CX43‐PARP1 axis. Neuron. 2023;111(22):3634‐3649.e7. 37683629 10.1016/j.neuron.2023.08.010

